# Research on the Behavioral Dynamics Motion Planning Method of the Human-Vehicle Social Force Model

**DOI:** 10.1155/2022/3154532

**Published:** 2022-10-28

**Authors:** Gaining Han, Zongsheng Wu, Wei Zhang, Wei Wang

**Affiliations:** School of Computer Science, Xianyang Normal University, Xianyang, Shaanxi 712000, China

## Abstract

The interactive motion planning between unmanned vehicles and pedestrians in urban road environments is the key to realizing the autonomous motion of unmanned vehicles in hybrid traffic scenarios. The problem of human-vehicle interaction motion planning modeling at complex intersections is studied for an unmanned vehicle in this article. First, the motion planning of pedestrians and the unmanned vehicles is established according to the social force model and the behavioral dynamics model. Then, the autonomous vehicle is added to the crowd, and the human-vehicle interaction force is established. The virtual force is added to the social force model and the behavioral dynamics model, respectively, and the improved social force model and the behavioral dynamics model are used for the motion planning of pedestrians and unmanned vehicles. In this way, the established model solves the problems of simple pedestrian interaction motion planning in the social force model and single-body motion planning in the behavioral dynamics and thus provides a strong support for multibody motion planning. Finally, through the interactive motion planning trajectory of pedestrians and unmanned vehicles in different scenes, the vehicle and pedestrian motion planning trajectory can effectively avoid overlapping or crossing, so as to avoid the collision, which verifies the effectiveness and feasibility of the proposed model.

## 1. Introduction

At present, the motion planning technology of unmanned vehicles (referred to as autonomous vehicles or self-driving vehicles) is mainly used to meet the obstacle avoidance function under vehicle and road constraints, as well as the requirements of multivehicle cooperation in the actual traffic environment and the intentions of other drivers [1–5]. However, autonomous vehicles will be sharing urban roads with human traffic participants for a long time in the future. Autonomous vehicles running on urban roads need to be able to autonomously interact with pedestrians and must be able to seize road resources [6–8]. Especially at urban intersections with complex traffic, the problem of human-vehicle interaction becomes more serious, making it difficult for autonomous vehicles to land and drive [9–12]. Therefore, it is very important to solve the problem of motion planning based on human-vehicle interaction.

The social force model (SFM) is an autodriven continuous model proposed by Helbing et al.,[13] which predicts the behavior and motion state of individuals in the later stage by analyzing individual physiological characteristics, desired speed, reaction time, individual distance, collision factors, and other conditions. The social force model mainly simulates individual and group movement according to Newton's second law formula, and the speed of the individual is compatible with the overall speed, which can portray the dynamic behavior of the individual. As a result, social force models are more used in areas such as emergency crowd evacuation [14–21], that is, most scholars have made many modifications and optimizations [22–30]. Jiang et al. [31] present a generalized walking cost distribution to determine a dynamic navigation field in the social force model for pedestrian evacuation and expect to choose an optimal path with the lowest walking cost to reach their target destination. The crowd evacuation simulation is performed using the density evacuation algorithm and the social force model and they can effectively improve the efficiency of crowd evacuation [32]. However, the social force model mainly is applied in the case of human-human interaction, rather than in scenarios of human-vehicle interaction [16, 33–35].

Yang et al. [16] present multipedestrian interaction with a low-speed vehicle based on a social force for pedestrian motion, a low-speed vehicle is not be considered the problem of motion planning, but it can be considered as a dynamic obstacle in the crowd. Wu et al. [36] develop a pedestrian heterogeneity-based social force model by introducing physique and mentality coefficients into the SFM to quantify the physiological and psychological attributes of pedestrians to change the desired speed and it does not consider the problem of human-vehicle interaction.

Unmanned vehicle motion planning problem, combining previous research results, and using the behavioral dynamics of attraction and repulsion to realize unmanned vehicle motion planning [37–39], this method uses virtual forces to control the heading angle and linear velocity of the unmanned vehicle and does not need to add empirical parameter adjustment control for path navigation, so as to reduce the accumulation of various empirical errors.

The aim of this research article is focused on the study of the motion planning problem of human-vehicle interaction and it combines the social force model and the behavioral dynamic model to realize unmanned vehicle motion planning during the two-way interaction between humans and vehicles. In the social force model, the unmanned vehicle is added to the crowd, the virtual interaction force between the autonomous vehicle and the pedestrian is introduced, called the human-vehicle force, and the social force model is used to plan the movement trajectory of the pedestrian. On this basis, the force received by pedestrians in the social force model is used as the repulsive force in the unmanned vehicle behavior dynamic model, and the motion planning of the unmanned vehicle is carried out.

The rest of the study is as follows: Section 2 introduces human-vehicle social force models and behavioral dynamics models, including the function of the social force model, virtual force analysis of pedestrians and unmanned vehicles, and modeling the behavior dynamics of the human-vehicle social force model. Constraints and conflict determinations are introduced in Section 3. Simulation results of illustration examples and discussion are presented in Section 4. Finally, in Section 5, the conclusion and further discussion are given.

## 2. Human-Vehicle Social Force Models and Behavioral Dynamics Models

### 2.1. Function of the Social Force Model

In the unmanned vehicle motion planning model, a process with such characteristics is required, when the obstacles around the unmanned vehicle show gradual and slow decay with the passage of direction and distance; then, this process is represented by the anisotropy function and attenuation function. The anisotropic function is used to describe the magnitude of the influence of interacting factors in different directions, for example, the vehicle or pedestrian in front of the unmanned vehicle has a significantly greater influence than the vehicle or pedestrian on its left or right; the attenuation function is used to describe the interaction results of different distances, for example, the vehicle or pedestrian who is far away from the unmanned vehicle has no influence on it, and the vehicle or pedestrian closer to the unmanned vehicle has a greater influence on it.

#### 2.1.1. Anisotropic Function

**Figure 1:**
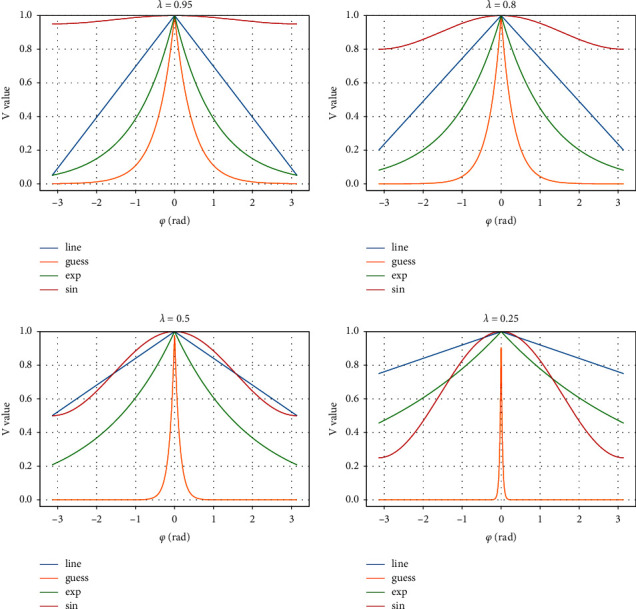
Different anisotropy functions.

The input and output representations of the anisotropic function [16], the angle between the direction of travel of the unmanned vehicle and the direction of motion of the interaction object as the input of the anisotropic function, and the output of the function is changed from 0 to 1, indicating that the anisotropy and function decrease with increasing. Commonly used functions are linear, exponential (exp), Gaussian (guess), and sin, and the four forms of anisotropic functions are shown in equations (1) to (4).

(1) $$\begin{array}{c} V_{line}\left(\varphi ,\lambda \right)=\max\;\left\{1-\lambda \frac{\left|\varphi \right|}{\pi },0\right\}\text{,} \end{array}$$

(2) $$\begin{array}{c} V_{guess}\left(\varphi ,\lambda \right)=\exp\;\left(-2*\frac{\left|\varphi \right|}{\lambda^{2}}\right)\text{,} \end{array}$$

(3) $$\begin{array}{c} V_{\exp}\left(\varphi ,\lambda \right)=\exp\;\left(-\lambda \left|\varphi \right|\right)\text{,} \end{array}$$

(4) $$\begin{array}{c} V_{\sin}\left(\varphi ,\lambda \right)=\lambda +\left(\left(1-\lambda \right)\frac{1+\sin\;\left|\varphi \right|}{2}\right)\text{,} \end{array}$$

where range change, which represents the angular change between the vehicle and the surrounding interacting objects, is the anisotropic adjustment factor. The different anisotropic function curves are shown in Figure 1.

As can be seen from Figure 1, when the *λ* value changes from 1 to 0, the main difference between these anisotropic functions is the decay rate near 0. When the *φ* value changes from −1 to +1, the Gaussian function decays the fastest, and the smaller the value of *λ* , the faster is the decay, while the sin function decays the slowest with the larger the value of *λ*. In this simulation test, the anisotropic function of *λ* = 0.8 is selected, and when the angle *φ* is [−*π*/3, +*π*/3], the Gaussian function is selected, and the *φ* between [−*π* × 3/2, +*π* × 3/2] is selected, the exponential or linear function is the exponential or linear function is selected; and the sine function is selected for other ranges. This variation plays an important role in the interaction between unmanned vehicles and surrounding objects.

#### 2.1.2. Attenuation Function

**Figure 2:**
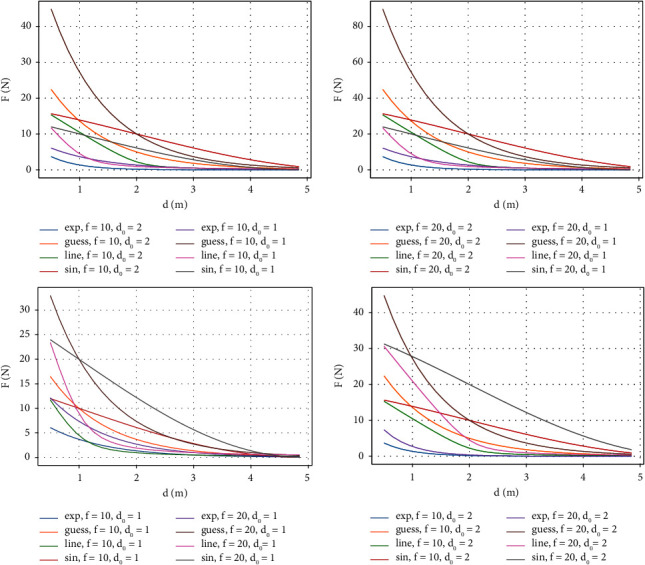
Different decaying functions.

The attenuation function is used to describe the influence on the unmanned vehicle at different distances. The distance between the unmanned vehicle and the interaction object is taken as the input, and the output of the function is the size of the virtual force, in unit N (Newton), which increases with the decrease of the distance. Similarly, linear, exponential, Gaussian, and sinusoidal functions are used to represent these four functions. This study compares these four functions through distance changes, and the function forms are shown in equations (5) to (9).

(5) $$\begin{array}{c} F_{\exp}\left(f,d,d_{0}\right)=f\;\exp\;\left({-d}_{0}*d\right)\text{,} \end{array}$$

(6) $$\begin{array}{c} F_{guess}\left(f,d,d_{0}\right)=\frac{f}{d_{0}}\exp\;\left(d_{0}-d\right)\text{,} \end{array}$$

(7) $$\begin{array}{c} F_{lin}\left(f,d,d_{0}\right)=\frac{f}{d_{0}}\left(d_{0}-d+\sqrt{d_{0}-d}+0.2\right)\text{,} \end{array}$$

(8) $$\begin{array}{c} F_{\sin}\left(f,d,d_{0}\right)=f\left(1+\left(\sin\frac{\left(d_{0}-d\right)}{2.5}\right)\right)\text{,} \end{array}$$

where *d* represents the change in the distance between the vehicle and the surrounding interaction object, *f* represents the virtual force at a distance of 0 when the distance between the vehicle and the surrounding interaction object is noncontact, and *d*_0_ is the set threshold distance. The change rules of the four functions under different *f* and *d*_0_ are shown in Figure 2.

As can be seen from Figure 2, when the values of *f* and *d*_0_ are different, these attenuation functions are mainly the change rate when the distance *d* between the vehicle and the interaction object approaches 0. The larger the value of *f*, the faster the decay. The higher the threshold *d*_0_ is, the faster will be the attenuation. Among the four functions, the guess function decays the fastest when *d* goes from 1 to 0. The attenuation rate is still exponential for the guess function, that is, when pedestrians approach unmanned vehicles, the attenuation function changes exponentially and acts as a human-vehicle interaction force. However, when pedestrians are far away from unmanned vehicles, they will not be affected by each other, so linear functions are selected as attenuation functions.

### 2.2. The Social Force Model

The social force model is based on Newton's second law, which reflects the relationship between the individual force and motion state, and according to the social force model [17], a single pedestrian of the total force includes a driving force towards the target, and an interaction between pedestrians, around and around obstacles between the force, and the basic expression of the model can be expressed as shown in the equation (9).

(9) $$\begin{array}{c} {\overset{⟶}{f}}_{i}\left(t\right)={\overset{⟶}{f}}_{itar}\left(t\right)+{\overset{⟶}{f}}_{ij}\left(t\right)+{\overset{⟶}{f}}_{iobs}\left(t\right)+{\overset{⟶}{f}}_{iego}\left(t\right)\text{.} \end{array}$$

This study mainly focuses on the interaction between pedestrians and ego vehicles, so the force ${\overset{⟶}{f}}_{iego}\left(t\right)$, between pedestrians and ego vehicles is added to the social force model. When it is added to the crowd, pedestrians will avoid ego vehicles. Therefore, vehicles also have a virtual “force” effect on pedestrians, which is called human-vehicle force in this study.

Due to the introduction of ego vehicles, pedestrians by force in addition to the driving force in the process of movement, from around pedestrians force and force from the obstacles around the outside, also suffered from the ego vehicles of force-car, therefore, on the basis of social force model, it will force people-car with three other kinds of forces in the form of vector addition, the vector and the combined force as a pedestrian, and then the relationship between mass, velocity, and the combined force is analyzed using Newton's second law. This social force model with human-vehicle interaction force is called the “human-vehicle social force model,” and its vector formula is shown in the following equation:

(10) $$\begin{array}{c} m_{i}\frac{d{\overset{⟶}{v}}_{i}\left(t\right)}{d\left(t\right)}={\overset{⟶}{f}}_{i}\left(t\right)={\overset{⟶}{f}}_{itar}\left(t\right)+{\overset{⟶}{f}}_{ij}\left(t\right)+{\overset{⟶}{f}}_{iobs}\left(t\right)+{\overset{⟶}{f}}_{iego}\left(t\right)\text{,} \end{array}$$

where *m*_*i*_ and *v*_*i*_ are the mass and speed of the *i* th pedestrian at a time *t*, ${\overset{⟶}{f}}_{i}\left(t\right)$ is the total acting force of the *i* th pedestrian at a time t, ${\overset{⟶}{f}}_{itar}\left(t\right)$ is the acting force running towards the target at a time *t*, ${\overset{⟶}{f}}_{ij}\left(t\right)$ is the interaction force between the *i* th pedestrian and the *i* th pedestrian at a time *t*, ${\overset{⟶}{f}}_{iobs}\left(t\right)$ is the interaction force at a time *t* with static obstacles, and ${\overset{⟶}{f}}_{iego}\left(t\right)$ is the interaction force at a time *t* with ego vehicle.

Pedestrian is a point mass in the social force model, and the position of the *i* th pedestrian is expressed as (*x*_*i*_, *y*_*i*_), and the speed is expressed as (*v*_*x*_*i*__, *v*_*y*_*i*__). According to Newton's law of motion, the following equation can be obtained.

(11) $$\begin{array}{c} \left\{\begin{array}{c} {\dot{x}}_{i}=v_{x_{i}},\\ {\dot{y}}_{i}=v_{y_{i}},\\ {\dot{v}}_{x_{i}}=a_{x_{i}}=\frac{F_{x_{i}}}{m_{i}},\\ {\dot{v}}_{y_{i}}=a_{y_{i}}=\frac{F_{y_{i}}}{m_{i}}. \end{array}\right. \end{array}$$

According to Newton's second law, the total force of the *i* th pedestrian at a time *t* is *f*_*i*_(*t*)=(*f*_*x*_*i*_,*t*_, *f*_*y*_*i*_,*t*_)^*T*^, and in Newton's law of motion, the *i* th pedestrian status information can be expressed as *X*_*i*_(*t*)=(*x*_*i*_(*t*), *y*_*i*_(*t*), *v*_*x*_*i*__(*t*), *v*_*y*_*i*__(*t*))^*T*^.

#### 2.2.1. Pedestrian-to-Pedestrian Interaction

The interaction force between pedestrians includes the total interaction force generated by all pedestrians around. According to the description of the interaction between pedestrians` in the original social force model, the interaction force between pedestrians is obtained by ignoring the friction of body extrusion and sliding. In practice, interaction forces between pedestrians include body collision force and virtual interaction force, as shown in the equation.

(12) $$\begin{array}{c} {\overset{⟶}{f}}_{ij}\left(t\right)=\underset{j\in \Omega \left(i\right)}{\sum }\left(f_{i,j}^{col}\left(t\right)+f_{i,j}^{vir}\left(t\right)\right)\text{,} \end{array}$$

where *j* ∈ Ω(*i*) represents the number of the other pedestrians around the *i* th pedestrian.

*f*_*i*,*j*_^col^(*t*) represents the force generated when pedestrian *i* and *j* are close to each other or their bodies are about to collide. The vector expression is shown in the formula.

(13) $$\begin{array}{c} f_{i,j}^{col}\left(t\right)=-A^{col}\;\min\;\left\{d^{i,j}\left(t\right),0\right\}{\vec{n}}^{ij}\text{,} \end{array}$$

where *A*^col^ is the collision coefficient and n^*ij*^ is the unit vector from pedestrian *i* to pedestrian *j*. When the boundary distance is reached, the repulsive force is generated, and the collision force is negative.

**Figure 3:**
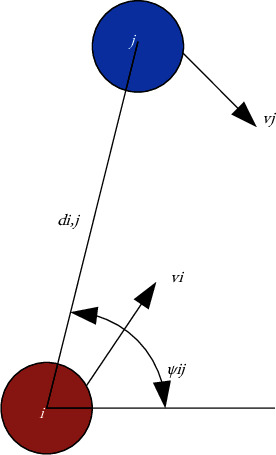
Schematic diagram of the movement of pedestrians i and j.

In formula (12), *f*_*i*,*j*_^vir^(*t*) is the virtual interaction force generated by the *i* th pedestrian and the *j* th pedestrian in a close distance. Current researchers have modeled the interaction force using circular rules, elliptic rules, obstacle avoidance rules, and exclusion navigation rules, which are based on the regional shape of the interaction range with or without speed and so on. In order to properly represent the effects of interaction forces, information such as position and velocity need to be considered to calculate the interaction force. Information such as position and velocity need to be considered to calculate interaction forces. The behavioral dynamics motion planning model adopts repulsive navigation rules, which are determined by repulsive force and azimuth angle, and can be expressed as shown in the following equations:

(14) $$\begin{array}{c} f_{i,j}^{vir}\left(t\right)=f_{i,j}^{rel}\left(t\right)+f_{i,j}^{\psi }\left(t\right)\text{,} \end{array}$$

(15) $$\begin{array}{c} \left\{\begin{array}{c} f_{i,j}^{rel}\left(t\right)=f_{i,j}\left(v_{i}\left(t\right),v_{j}\left(t\right),d_{i,j}\left(t\right)\right)\text{,}\\ f_{i,j}^{\psi }\left(t\right)=f_{i,j\_\psi }\left(\psi_{i,j}\left(t\right)\right)\text{,} \end{array}\right. \end{array}$$

where *ψ*_*i*,*j*_(*t*) is the included angle between the line direction from pedestrian *i* to pedestrian *j* and the horizontal direction, as shown in Figure 3.

#### 2.2.2. Pedestrian and Vehicle Interaction

**Figure 4:**
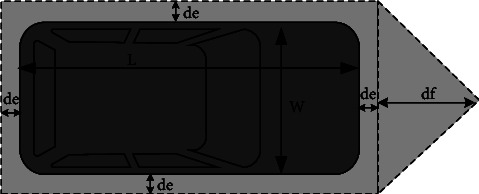
Schematic diagram of vehicle safety range.

Vehicle-pedestrian interactions are different from pedestrian-pedestrian interactions in which collisions between pedestrians and vehicles are strictly prohibited. The relative position and speed parameters between pedestrian and vehicle, size and shape of vehicle, and anisotropy are considered in order to give the vehicle a stronger force range. Thus, different pedestrian directions and speeds as well as different vehicle speed directions lead to different interactions between vehicles and pedestrians. At the same time, it is important to establish a safety buffer area around the vehicle, that is, the minimum distance between pedestrians and vehicles, as shown in the gray dotted box in Figure 4.

In Figure 4, *df* is the buffer length along the driving direction of the vehicle and *df*=*kv*_*x*_ is proportional to the longitudinal speed of the vehicle. The faster the speed of the vehicle, the longer is the buffer *df* for in front of the vehicle. The *de* is the buffer range of vehicle obstacle avoidance. After the buffer area is determined, the interaction force is determined by considering the minimum distance between pedestrians and buffer, anisotropy and attenuation, and the interaction force between vehicles and pedestrians is calculated by following formula.

(16) $$\begin{array}{c} {{\overset{⟶}{f}}_{iego}\left(t\right)=f_{guess}\left(d\right.}_{i,ego}\left(t\right),{A\;}^{ego},\left.B^{ego}\right)V\left(\varphi ,\lambda^{ego}\right){\vec{n}}^{iego}\text{,} \end{array}$$

where *f*^ego^ and *B*^ego^ are the coefficients of the attenuation function. *λ*^ego^ is the coefficient of the anisotropy function, *φ* is the angle between pedestrian and ego vehicle, *d*_*i*,ego_ is the distance between pedestrian and ego vehicle, and ${\vec{n}}^{iego}$ is the unit vector between pedestrian and ego vehicle, when there is no unit.

#### 2.2.3. The Driving Force to the Goal

When the pedestrian is disturbed by the surrounding pedestrians and the environment, the pedestrian's speed decreases and it generally becomes lower than its expected speed. In order to achieve the desired speed as soon as possible, the pedestrian will try to maintain the desired speed throughout the automatic driving force. The driving force towards the target can be expressed as follows.

(17) $$\begin{array}{c} f_{i}^{tar}\left(t\right)=f\left(v_{i}^{tar}\left(t\right),v_{i}\left(t\right)\right)=k_{i}^{tar}\beta_{i}^{tar}\left(v_{i}^{tar}\left(t\right)-v_{i}\left(t\right)\right){\vec{n}}^{itar}\text{,} \end{array}$$

where *k*_*i*_^tar^ is the coefficient of the driving force. *β*_*i*_^tar^ ∈ [0,1], when the pedestrian-vehicle interaction force reaches a certain set threshold, its value is 1, otherwise it is 0, as shown in equation (18), *v*_*i*_^tar^ is the expected pedestrian speed in m/s, *v*_*i*_(*t*) is the actual pedestrian speed in m/s, and ${\vec{n}}^{itar}$ is the unit vector of the direction of the expected speed, without a unit.

(18) $$\begin{array}{c} \beta_{i}^{tar}=\max\;\left\{0,\min\;\left\{\frac{\left|f_{iego}\left(t\right)\right|}{F_{1}},1\right\}\right\}\text{,} \end{array}$$

where *F*1 is the setting threshold.

#### 2.2.4. Dynamic Model of Vehicle Behavior

In order to make the designed model to be effectively applied in the actual motion planning and in the control of the ego vehicle, that is, the basis on the “pedestrian-vehicle social force model”, the vehicle movement adopts the behavioral dynamics model and it is adopted so as to ensure that the ego vehicle can drive normally in the external environment. The obstacle avoidance behavior in the behavioral dynamics is combined with the pedestrian-vehicle interaction force in the social force model, so as to realize the effective interaction model of “pedestrian-vehicle” and “pedestrian-pedestrian,” and to improve the single interaction model centered on “pedestrian” under the social force model.

According to the characteristics of the behavior pattern, the virtual force generated by the behavior of running towards the target is called attraction, and the virtual force generated by the behavior of avoiding obstacles is called repulsion. Attraction guides the vehicle along a certain direction towards the target point, thus generating driving speed and direction of the vehicle. Repulsion prevents the vehicle from approaching its obstacle and causes the vehicle to travel in a direction away from the obstacle. Repulsion increases with decreasing distance and also creates a speed and direction that prevents the vehicle from traveling toward the obstacle. According to the ego vehicles by virtual forces, including towards the goal of the driving force, with the surrounding traffic participants and the interaction between road obstacles such as the boundary, can be established based on Newton's law *mv*=*f*_*v*_(∙) and behavior dynamics model, and it reflects the relationship between the force and motion state of ego vehicles. The basic expression is shown in the formula.

(19) $$\begin{array}{c} \overset{⟶}{F}\left(t\right)={\overset{⟶}{F}}_{tar}\left(t\right)+\overset{N}{\underset{i=1}{\sum }}{\overset{⟶}{F}}_{obs,i}\left(t\right)\text{,} \end{array}$$

where ${\overset{⟶}{F}}_{tar}\left(t\right)$ is the attraction of running towards the target at a time t, and ${\overset{⟶}{F}}_{obs,i}\left(t\right)$ is the repulsive force generated by the first obstacle at a time *t*. When there are multiple obstacles, the resultant force should be calculated first.

Assuming that the ego vehicle is driving at ${\overset{⟶}{v}}_{\text{ego}}\left(t\right)$ , then it should drive at a desired speed ${\overset{⟶}{v}}_{tar}\left(t\right)$ as much as possible. In actual driving, there is a certain deviation between ${\overset{⟶}{v}}_{ego}\left(t\right)$ and ${\overset{⟶}{v}}_{tar}\left(t\right)$ at a time *t*, and the virtual force of speed attraction can drive the vehicle to change the driving speed, and the force of speed attraction is proportional to $\left({\overset{⟶}{v}}_{\text{ego}}\left(t\right)-{\overset{⟶}{v}}_{tar}\left(t\right)\right)$. If ${\overset{⟶}{v}}_{\text{ego}}\left(t\right)={\overset{⟶}{v}}_{tar}\left(t\right)$, then the attraction is 0 and the vehicle keeps the current speed. The force of speed attraction at the moment can be expressed as.

(20) $$\begin{array}{c} {{\overset{⟶}{F}}_{tar}\left(t\right)=f\left(\overset{⟶}{v}\right.}_{\text{ego}}\left(t\right)-{\overset{⟶}{v}}_{tar}\left.\left(t\right)\right)\text{.} \end{array}$$

In the process of driving, other traffic participants within the perspective range of ego vehicle will interfere with it, thus forming a repulsive force. The farther the distance from obstacles, the smaller the repulsive force. Thus, the amount of repulsive force depends on the current number of obstacles in space (i. e., density) and the current speed of the vehicle, which increases as the distance decreases. The repulsive force at the moment can be expressed by the following equation.

(21) $$\begin{array}{c} {\overset{⟶}{F}}_{obs,i}\left(t\right)=\left\{\begin{array}{c} f\left({\overset{⟶}{v}}_{ego}\left(t\right),{\overset{⟶}{v}}_{obs,i}\left(t\right),d_{obs,i}\right),\\ 0, \end{array}\right.\;\begin{array}{c} d_{obs,i}<d_{s},\\ d_{obs,i}>d_{s}, \end{array} \end{array}$$

where the speed of the first obstacle, if the obstacle is stationary, *v*_obs,*i*_(*t*) is 0, *d*_obs,*i*_ is the distance between ego vehicle and the *i* th obstacle, and *d*_*s*_ is the safe driving distance. Specific derivation and calculation processes can be referred to in literatures [37–39].

#### 2.2.5. Behavioral Dynamics Model of the Human-Vehicle Social Force Model

The behavior dynamics model of the human-vehicle social force model (FSM-BDM for short) introduces the human-vehicle interaction force into the social force model and into the navigation behavior dynamics model, the navigation behavior dynamics motion planning model adopts navigation rules based on repulsive force, where ${\overset{⟶}{f}}_{iego}\left(t\right)$ is the repulsive force generated as an obstacle. Thus, two system models about people and ego vehicles are established, as shown in equation (22).

(22) $$\begin{array}{c} \left\{\begin{array}{c} {\overset{⟶}{f}}_{i}\left(t\right)={\overset{⟶}{f}}_{itar}\left(t\right)+{\overset{⟶}{f}}_{ij}\left(t\right)+{\overset{⟶}{f}}_{iobs}\left(t\right){+\overset{⟶}{f}}_{iego}\left(t\right)\text{,}\\ \overset{⟶}{F}\left(t\right)={\overset{⟶}{F}}_{tar}\left(t\right)+\overset{N}{\underset{i=1}{\sum }}{\overset{⟶}{F}}_{obs,i}\left(t\right){+\overset{⟶}{f}}_{iego}\left(t\right)\text{.} \end{array}\right. \end{array}$$

The obstacle avoidance behavior in behavioral dynamics is combined with human-vehicle interaction force in the social force model, so as to realize the effective two-way interaction model of “pedestrian-vehicle” and “pedestrian-pedestrian,” and to improve the single interaction mode centered on “pedestrian” under the social force model.

### 2.3. Constraints and the Model Parameter Settings

#### 2.3.1. Constraint Analysis

Considering that pedestrians and vehicles are constrained by the road and the surrounding environment in the process of movement, there are certain limitations in speed and acceleration. First of all, pedestrians move in a way for the purpose of comfort under normal circumstances. Unless there is an emergency, for example, if a vehicle approaches pedestrians in a dangerous way, pedestrians will slow down or stop for self-protection. For example, when pedestrians are crowded, they naturally slow down. Second, vehicle traffic on the road, according to the road environment will speed up, slow down or stop, therefore, exertion on the pedestrian and vehicle speed and acceleration constraints are related to time, and at the same time, the constraint conditions need to consider the interaction between vehicles and pedestrians *f*_iego_(*t*) and pedestrians near I sparse degree *D*_*i*_(*t*), as shown in the equation.

(23) $$\begin{array}{c} \left\{\begin{array}{c} v_{i}\left(t\right)\leq v_{\lim}\left(f_{iego}\left(t\right),D_{i}\left(t\right)\right)\text{,}\\ a\left(t\right)\leq a_{\lim}\left(f_{iego}\left(t\right),D_{i}\left(t\right)\right)\text{,}\\ D_{i}\left(t\right)=\min\;\left(\frac{N}{\left|S_{t}\right|}\;V\left(\varphi^{ij}\left(t\right),\lambda \right)\right),\forall j\in S_{t}\text{,} \end{array}\right. \end{array}$$

where *D*_*i*_(*t*) is the sparsity of the *i* th pedestrian, *N* is the number of pedestrians within the limited range, *S*_*t*_ is the measured area of the limited area, and *V*(*φ*^*ij*^(*t*), *λ*) is the anisotropy function.

#### 2.3.2. Constraint Set

*(1) Pedestrian speed and acceleration constraints*. According to the literature [17, 18, 36], when pedestrians walk on flat roads, the maximum walking speed limit is *v*_max_=2.5m/s, the normal walking speed limit is *v*_nor_=1.8m/s, the density speed limit is *v*_den_=0.7m/s, the maximum walking acceleration is *a*_max_=5m/s^2^, the normal walking acceleration is *a*_nor_=2.5m/s^2^, and the density acceleration is *a*_den_=0.1m/s^2^.

*(2) Constraints on vehicle velocity and acceleration*. In order to maintain driving comfort, the vehicle speed and acceleration are limited *v*_min_=0m/s, *v*_max_=*v*_limt_m/s, and *v*_limt_ is the actual maximum speed limit on the road. Acceleration is divided into deceleration and acceleration, which are *a*_min_=−1.5m/s^2^ and *a*_max_=1.5m/s^2^.

Table 1 describes other parameters of people-vehicle social force model.

#### 2.3.3. Ego Vehicle and Obstacle Conflict Determination

We determine whether there will be a conflict between the ego vehicle and the obstacle in the future. If there is a conflict, then we calculate the direction and magnitude of the force exerted by the obstacle on the ego vehicle, as follows:

*(1) Determine whether there will be conflicts between ego vehicles and obstacles*. When the ego vehicle detects an obstacle while driving, the ego vehicle starts to judge whether it will collide with the obstacle or not. The current speed of the ego vehicle is *v*_ego_, and its actual speed at a ∆*t* time can be calculated according to the formula.

(24) $$\begin{array}{c} v_{ego}^{des}=v_{ego}+\frac{f_{ego}}{m_{ego}}∆t\text{.} \end{array}$$

The position of the ego vehicle after ∆*t* time is obtained by the following formula. The time *t* value can be obtained by combining the *y*_ego_^des^ coordinate with the obstacle position coordinate.

(25) $$\begin{array}{c} y_{ego}^{des}=y_{ego}+v_{ego}^{des}t\text{.} \end{array}$$

1. When *t* has no solution, there will be no collision with the obstacle.
2. When *t*_1_=*t*_2_ is a plural, the vehicle will collide with the obstacle.
3. When *t*_1_=−*t*_2_ ≠ 0, *t*_1_ and *t*_2_ are opposite, and the vehicle will collide with the obstacle, and *t* becomes greater than 0.

By judging that the ego vehicle will not collide with the obstacle, if it is judged that a conflict will occur, the conflict time is denoted as *t*_*aw*_.

*(2) According to the time of conflict, obstacles with force on the unmanned vehicle are selected*. Since there are not many obstacles even in a complex environment, it is decided to select all obstacles that meet the conflict time for force calculation. We elect obstacles according to the rule that *t*_*aw*_ is less than *T*_2_ seconds (*T*_2_ = 3 s to be calibrated).

1. When *0* *≤* *t*_*aw*_ *<* *T*_2_ second, all obstacles within this time range are selected for force calculation.
2. When *t*_*aw*_ *>* *T*_2_ second, the influence of these obstacles on the ego vehicle is not considered.

## 3. Experimental Simulation and Analysis

To verify and test the abovementioned SFM-BDM, this part first experiment showed ego vehicles and pedestrian conflict, and obstacles of decision analysis. By setting different traffic scenarios, we test the pedestrian and vehicle interaction under this model, through the pedestrian trajectory, pedestrians and vehicles and vehicle velocity and acceleration analysis, and the validity and feasibility of the model.

### 3.1. Analysis of Human-Vehicle Interaction Scenarios

**Figure 5:**
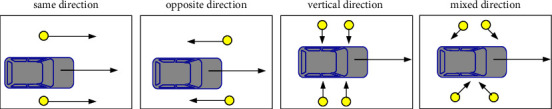
Pedestrian-vehicle interaction for four typical scenarios.

According to the analysis of actual vehicle-human interaction traffic scenarios, vehicle-human interaction is mainly divided into parallel and crossover scenarios. Parallel scenarios include driving in the same direction and driving in the opposite direction, and crossover scenarios include vertical and mixed driving, as shown in Figure 5.

Same direction, opposite direction, vertical direction, and mixed direction.

### 3.2. Human-Vehicle Interaction Scenario Test

#### 3.2.1. Ego Vehicle Stationary

**Figure 6:**
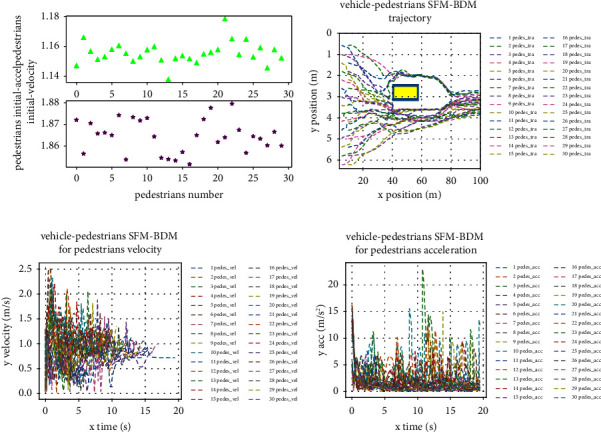
Pedestrian and vehicle interaction scene when the vehicle is stationary. (a) Pedestrian initial velocity and acceleration. (b) Pedestrian's trajectory. (c) Pedestrian's velocity. (d) Pedestrian's acceleration.

When the ego vehicle is static, SFM-BDM the motion planning problem of is converted into a pedestrian motion planning problem, the scene is set to have 30 pedestrians through a stationary vehicle, pedestrian's initial position is on the left side of the scene; velocity and acceleration by the Monte Carlo algorithm; pedestrians target is right at the scene of the middle area. We test the motion planning trajectory of pedestrian and stationary vehicles under SFM-BDM, as shown in Figure 6.

#### 3.2.2. Pedestrian and Vehicle Movement Test in the Same Direction

**Figure 7:**
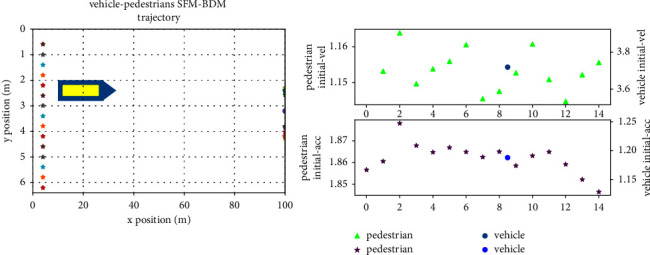
Initialization scene of pedestrians and vehicles in the same direction.

When the movement scene of pedestrians and vehicles in the same direction is bidirectional single-lane or one-way single-lane, pedestrians and vehicles move in the same direction when driving. Assuming that the test scene of driving in the same direction is set as 15 pedestrians and one ego vehicle. Under FSM-BDM, the initialization scene is shown in Figure 7, which includes the initial position and target position of pedestrians and ego vehicles and also includes the initial velocity and acceleration.

**Figure 8:**
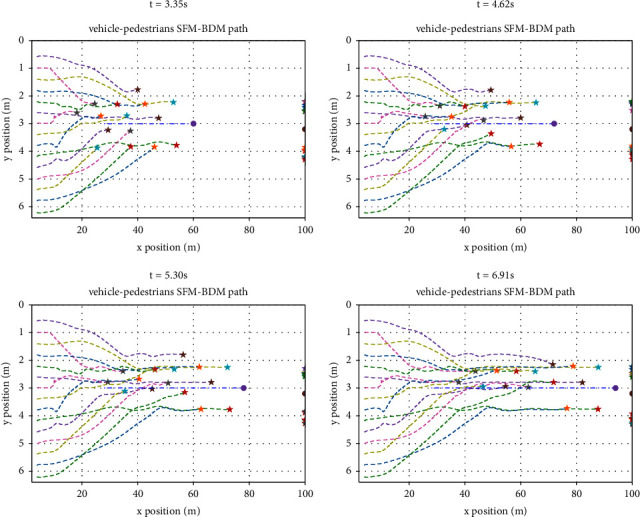
Pedestrians and vehicles moving in the same direction.

Figure 8 shows the movement planning tracks of pedestrians and vehicles at different times. This scene tests the mixed movement of humans and vehicles. In the case of a large number of pedestrians, ego vehicles run towards the target according to the behavioral dynamics model, and each pedestrian moves and runs towards the target under the social force model. The blue dotted line in the figure represents the trajectory of ego vehicle movement planning, while the other dotted lines represent the trajectory of pedestrians. Since the target points of pedestrians are different, pedestrians will move along the nearest distance when it is safe. Under this model, pedestrians and ego vehicles can effectively pass safely and run to their respective targets.

#### 3.2.3. Pedestrian and the Vehicle Reverse Motion Test

**Figure 9:**
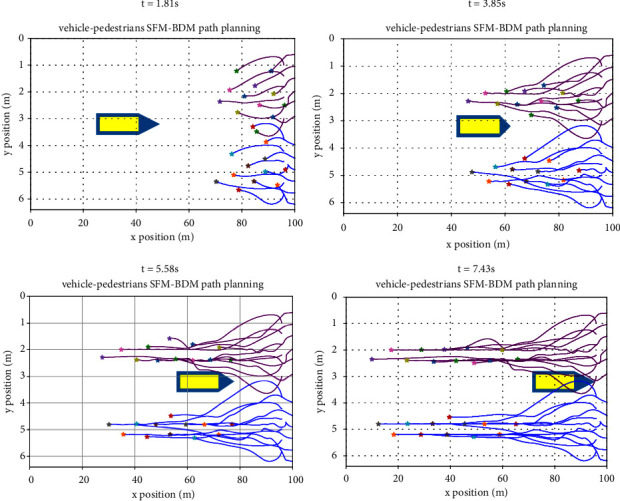
Pedestrian and vehicle reverse interaction scene.

In Figure 9, the reverse movement of pedestrians and vehicles is tested. Ego vehicles run in reverse in a crowd with 12 pedestrians on both sides. The movement tracks of pedestrians and ego vehicles at different times are shown in the figure. Among them, the blue and purple path is for random pedestrians on both sides, yellow for ego vehicles, yellow blue area is the ego around vehicle safety area, ego in the process of the vehicle, the triangle area and the vehicle in front of the longitudinal velocity is proportional to the buffer area, when pedestrians and ego vehicles without interaction, because the ego vehicle speed, so the buffer is big. When there is an interaction, the ego vehicle speed decreases, so the buffer becomes smaller, especially when *t* = 3.85 s, it is in the interaction range. Therefore, under the human-vehicle social force model, pedestrians and ego vehicles can effectively and safely avoid obstacles.

#### 3.2.4. Pedestrian and the Vehicle Mixed Motion Test

**Figure 10:**
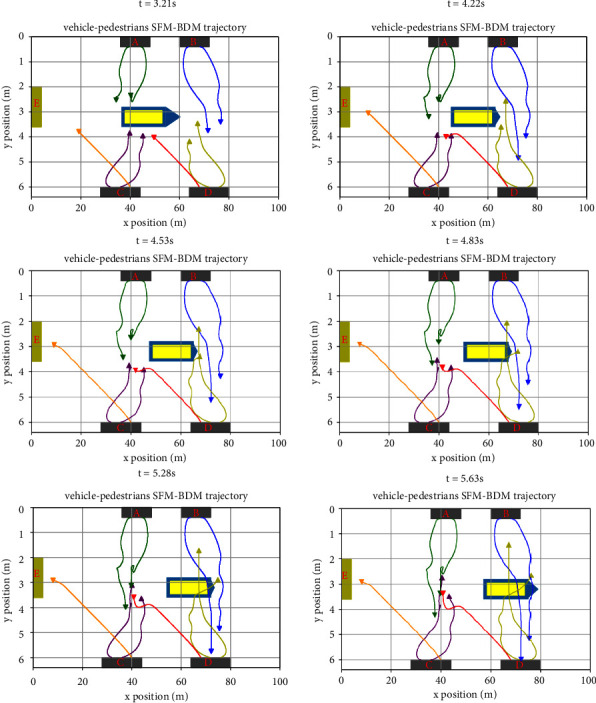
Pedestrian and vehicle mixed interaction scenario.

The test scenario of mixed driving of pedestrians and vehicles shown are in Figures 4–10, and also, the running tracks of pedestrians and the moving positions of ego vehicles at different times are respectively shown in the figure. The test scenario is similar to the pedestrian crossing lane, and different places are set up on both sides of the lane, which are represented by gray rectangular areas in the figure and are marked as (A, B, C, and D) respectively. On the left are traffic exits, in which the yellow-green rectangular area are marked as E. Yellow rectangles for ego vehicles are driven from left to right, respectively, on the road on both sides of the set for different number of pedestrians, the figure in the green line represents the pedestrian from position A to position the trajectory of C, the blue line represents the pedestrian from location to location D B trajectory, lemon line pedestrians from location to location B D trajectory, purple line represents the pedestrian from C to the location of A trajectory, The orange line represents the path of the pedestrian from position C to position E, and the red line represents the path of the pedestrian from position D to position A. Under SFM-BDM, the trajectory of pedestrian and vehicle motion planning at different times is shown in Figure 10.

Figure 10 shows the interaction between pedestrians and includes the interaction between pedestrians in the same direction and pedestrians on the opposite side, and there is no collision between pedestrians or pedestrians and vehicles. In Figure 10, between 4.22 s and 5.63 s, pedestrians and vehicles interact most closely. At this point, under the action of human-human interaction force and human-vehicle interaction force, pedestrians can randomly change their walking status and can achieve an obvious obstacle avoidance effect.

**Figure 11:**
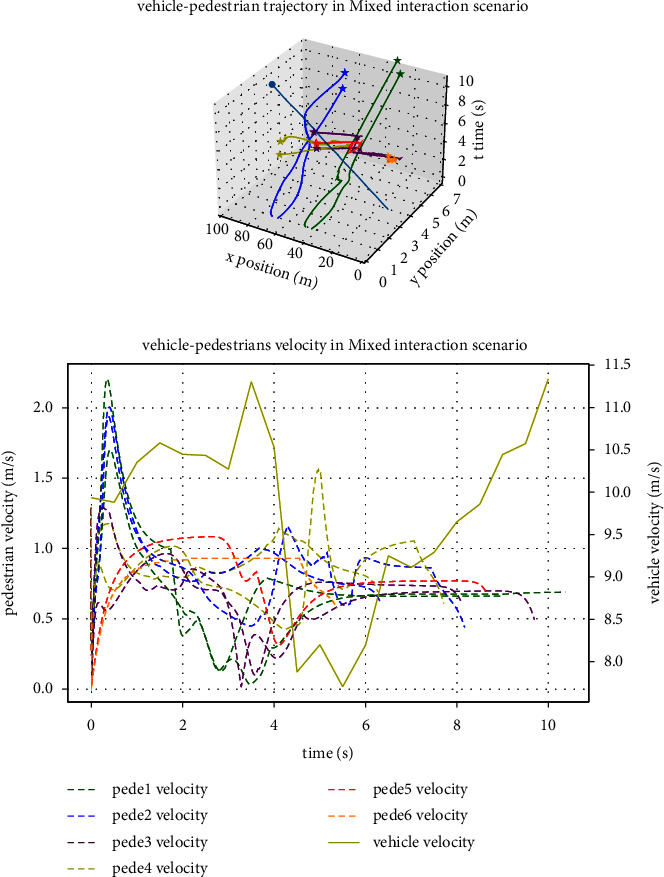
Pedestrian and vehicle mixed interaction motion trajectory and velocity: (a) motion trajectory and (b) vehicle and pedestrian's velocity.

Figure 11 for vehicle and pedestrains trajectory and velocity from Figure 10, Figure 11(a) shows pedestrians and vehicle on the x and y coordinates change with time in the process of movement. Through the analysis of position and time, it can show that the model can make pedestrians and pedestrians, pedestrians and vehicle repel and avoid obstacles behavior in the process of movement. There is no overlap in space positions which is without collision.  Figure 11(b) shows the speed of pedestrians and vehicles. When pedestrians and ego vehicles interact closely between 4 s and 5.6 s, there is a process of deceleration and avoidance due to the close interaction. After obstacle avoidance, pedestrians and ego vehicles move towards their respective target positions at the expected speed.

## 4. Conclusion

This study is mainly based on the research of movement methods of the human-vehicle social force model and the behavior dynamics model.

1. The behavioral dynamics motion planning method of the constructed “human-vehicle” social force model introduces the human-vehicle interaction into the social force model and the behavioral dynamics model and solves the motion planning problem by considering the human-vehicle two-way interaction.
2. The behavioral dynamics motion planning method of the “human-vehicle” social force model solves the motion planning of the interaction between multiple people and unmanned vehicles, which can be further extended to the motion planning of the interaction between multiple cars and multiple people, and the motion planning considering the simultaneous change of the speed and direction of unmanned vehicles.
3. The proposed motion method based on the pedestrians-vehicle social force model and behavior dynamics was simulated in different scenarios to verify the safety and effectiveness of the proposed method in the process of human-vehicle interaction. The research in this chapter effectively combines behavioral dynamics motion planning with the social force model, which is more in line with the actual scene, provides an adaptive motion planning for ego vehicles, and solves the process of pedestrians-vehicle interaction motion planning by relying on a large number of scene data learning.
4. This method can provide support for the interactive obstacle avoidance and effective motion planning of unmanned vehicles in the urban road environment and can lay the foundation for the next tracking control.

## Figures and Tables

**Table 1:** People-vehicle social force model parameters.

Parameter	Value	Description	Unit
L	2.4	Vehicle length	m
W	1.2	Vehicle width	m
*d*_*e*_	0.20	Offset distance around the vehicle	m
*m*_vech_	1120	Vehicle quality	kg
*v*_veh_^des^	11	Expected speed	m/s
*l*_*t*_	0.8	Weight coefficient of toward target	
*l*_*o*_	0.2	Weight coefficient of obstacle avoidance	
*A* ^veh^	800	Initial value of the coefficient of anisotropic	
*B* ^veh^	−0.82	Initial value of the coefficient of anisotropic	
*λ* ^veh^	0.32	Initial coefficient of the attenuation	
*F*_1_	400	Threshold value	N
*m*_ped_	[40 ~ 70]	Quality of pedestrian	kg
*r*_ped_	[35 ~ 40]/2/100	Radius of pedestrian	m
*v*_ped_^des^	[0.7 ~ 1.8]	Expected pedestrian speed	m/s
*a*_ped_^des^	[0.1 ~ 2.5]	Expected pedestrian acceleration	m/s^2^
num_ped_	[1 ~ 45]	Number of pedestrian	

## Data Availability

The data supporting the findings of this study are available from the corresponding author upon reasonable request. The nature of the data is algorithm programs.
